# Hoverflies of the Timon-David collection (Diptera, Syrphidae)

**DOI:** 10.3897/BDJ.12.e117265

**Published:** 2024-02-19

**Authors:** Gabriel Nève, Xavier Lair, Thomas Lebard, Jean-Yves Meunier, Louis-Jean Teste, Louise Séguinel

**Affiliations:** 1 IMBE, Marseille, France IMBE Marseille France; 2 Aix Marseille University, Marseille, France Aix Marseille University Marseille France; 3 CNRS, Marseille, France CNRS Marseille France; 4 IRD, Marseille, France IRD Marseille France; 5 Avignon University, Avignon, France Avignon University Avignon France; 6 Independent Researcher, Sournia, France Independent Researcher Sournia France; 7 Independent Researcher, Breil-sur-Roya, France Independent Researcher Breil-sur-Roya France; 8 MHNM, Marseille, France MHNM Marseille France

**Keywords:** Jean Timon-David, Museum collection, distribution, France, Diptera, Syrphidae, pollinators

## Abstract

**Background:**

Hoverflies are among the most important insect pollinators and there is documented evidence of a recent decline in their populations. To trace the past distributions of hoverfly species, verified records of historical collections are essential.

**New information:**

Here, we provide data on 1071 specimens of hoverflies collected or received by Jean Timon-David and hosted at the Marseille Natural History Museum, France. Most of the specimens were collected by Timon-David himself and come from south-eastern France, mainly from the Departments of Bouches-du-Rhône, Var and Hautes-Alpes. Most of these specimens were checked for the accuracy of their identification according to the latest identification keys. This resulted in 85 additions to the known fauna of the French Departments, mostly for Var and Bouches-du-Rhône. The taxonomy of all specimens was checked against the latest available checklists and updated names added whenever necessary. Specimens received from entomologists working in other continents may also be valuable, as these are historic testimonies of the fauna of their own respective regions of origin and may, therefore, also be used as reference material. One paratype specimen from Australia is present in the collection. The holotype of *Cheilosia vangaveri* Timon-David, 1937 is absent from the collection and should be considered as lost. All but two of the specimens with locality labels had their geographical coordinates of origin added in the dataset.

## Introduction

Hoverflies (Diptera, Syrphidae) are known to be among the most important pollinators of flowering plants, along with Hymenoptera, Lepidoptera and Coleoptera (Ssymank et al. 2008, Chisausky et al. 2020). The documentation of past distribution concerning these insects relies on existing collections, where the identification of specimens can be checked, in order to document changes in the distribution and relative abundance of the various species.

The aim of the present paper is to record the important hoverfly collection hosted at the Marseille Natural History Museum, contributing to the endeavour to document the past distribution of pollinators in France (Meunier et al. 2023, Zakardjian et al. 2023).

### Historical background

**Figure 1.**
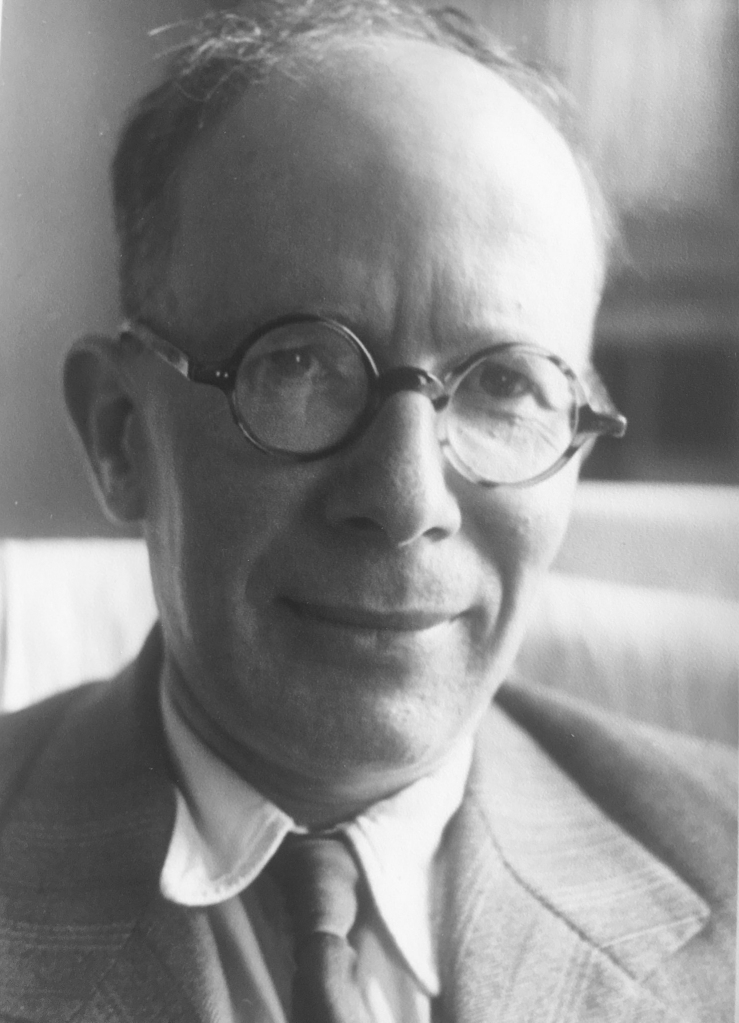
Portrait of Jean Timon-David (photograph supplied by Pierre Timon-David).

Jean Timon-David (Fig. 1) was born in Marseille on 3 September 1902 and died there on 20 November 1968 (Nicoli and Timon-David 1973). As a professor of zoology and parasitology, Timon-David was described by one of his former students as a short and shy man whose lectures showed a wide and deep knowledge of his subject (M.-T. Cordier, pers. comm. 2020). He was both a conscientious researcher and a dedicated teacher, much loved by his students (Dollfus 1969). His reserved personality may explain why his academic career was slow: Jean Timon-David was both a doctor of medicine (at Montpellier in 1926) and a doctor of science (at Sorbonne in 1930). He was appointed Head of Zoology, *Faculté des Sciences de Marseille*, at the age of 35, Senior Lecturer at 50 and full Professor of Zoology at 58. It seems that his shy temperament did him a disservice in his career development. On the other hand, his great scientific skills were recognised in scientific circles: he was *Officier d'Académie, Officier de l'Instruction Publique* and *Chevalier du Mérite Agricole* (Nicoli and Timon-David 1973). In entomological circles, he was internationally recognised by his peers. Hull (1973) mentions “the late Professor J. Timon-David of Marseille” in his acknowledgements at the beginning of his monograph of the Bombylidae of the world alongside some the best known dipterists of the XX^th^ century “Dr. Willi Hennig [1913-1976], Dr. Erwin Lindner [1888-1988] and the late Professor Fred Keiser [1895-1969]”. In addition to entomology, Jean Timon-David's work covered geology, biochemistry (his doctoral thesis was on insect fats), ichthyology, ornithology and, above all, parasitology. A tribute to his research in parasitology was published in 1968 in the *Annales de Parasitologie* (Dollfus 1968) and a scientific biography with a full bibliography was published in 1973 (Nicoli and Timon-David 1973).

### Jean Timon-David’s contribution to entomology

Jean Timon-David collected insects, mainly Diptera and Hymenoptera, from 1926 to 1961, and published pioneering research on the Diptera of the Mediterranean islands off the French coast (Van Gaver and Timon-David 1929a, [Bibr B10912785][Bibr B10912772], Timon-David 1940, Timon-David 1961), the high Alps (Van Gaver and Timon-David 1929b, Timon-David 1931, Timon-David 1937a, Timon-David 1937b), the Pyrenees (Timon-David 1949, Timon-David 1950) and the Sainte-Baume Massif (Timon-David 1936, Timon-David 1958). He also revised Diptera material from Morocco (Timon-David 1951a). Timon-David was a recognised specialist of Asilidae (Diptera) and his collection includes 18 boxes of material belonging to this family, with specimens from 13 different countries, including one box from the agricultural station at Boukoko (Oubangi-Chari, Central African Republic). He published an important series of revisions on the Asilidae of Madagascar (Timon-David 1951b, Timon-David 1952, Timon-David 1953a, Timon-David 1953b), with the description of several new species. One species of Asilidae from the Camargue was dedicated to him: *Heteropogon timondavidi* Tsacas 1970.

Indirectly, the labels of the specimens in his Syrphidae collection also show the regular contact he had with his foreign colleagues. For example, Ralph Leonard Coe (British Museum (Natural History)) identified some of his specimens. Timon-David received specimens from Charles Rungs (four Moroccan specimens), Frank M. Hull (U.S.A.), H.L. Lopes (Brazil), L. Richter (Colombia) and Edgar F. Riek (Australia).

As Nicoli and Timon-David (1973) point out, Jean Timon-David's collections from around Marseille date from a time when these areas were not yet fully urbanised. Thus, examination of this particular collection is a great opportunity for researchers to open a window into the past of this region which has since then been totally transformed and gives precious information about its environment which cannot be obtained by any other means.

## General description

### Purpose

The aim of this publication is to record all of the Syrphidae specimens hosted in the Timon-David collection at the Muséum d'Histoire Naturelle de Marseille (MHNM).

**Figure 2.**
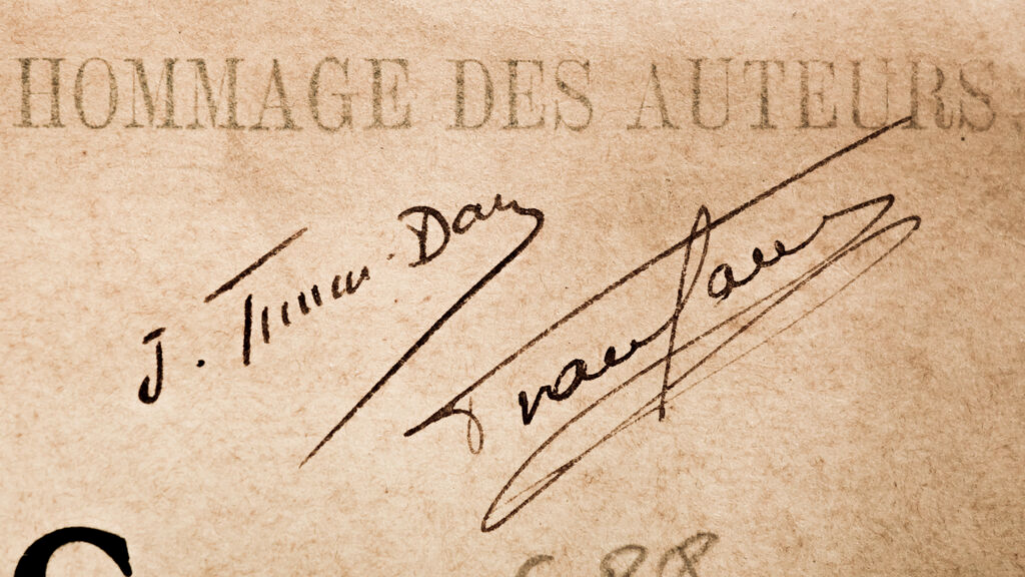
Signatures of Jean Timon-David and Ferdinand Van Gaver on the cover of their joint 1937 publication (Van Gaver and Timon-David 1937), now in the Saint-Charles University Library, Aix Marseille University.

We also want to raise awareness of the entomological work of Jean Timon-David, whose publications, in French, were often in regional journals or conference proceedings and, therefore, difficult to access. In their recent synthesis of the Syrphid fauna of France, Speight et al. (2018) cite only one of Timon-David's publications, that of 1937 which contains the original description of *Cheilosia vangaveri* (Timon-David, 1937), named in honour of Ferdinand Van Gaver (1874-1943), the only colleague with whom Timon-David published on Syrphidae (Van Gaver and Timon-David 1928, Van Gaver and Timon-David 1929a, Van Gaver and Timon-David 1929b). A signed reprint of one of their joint publications (Van Gaver and Timon-David 1937) was given to the University Library (Fig. 2).

The entomological collection of Jean Timon-David was given to the Muséum d’Histoire Naturelle de Marseille in 2006 by his son Pierre Timon-David. It contains a total of 114 boxes, including about ten thousand specimens. The aim of the present paper is to publish all the data on Syrphidae from this collection as a tribute to Jean Timon-David’s outstanding work in entomology.

The identification of most specimens from France has been checked recently and the taxonomy of the whole collection has been brought up-to-date.

## Project description

### Title

Timon-David collection of Syrphidae (Diptera)

## Sampling methods

### Study extent

**Figure 3.**
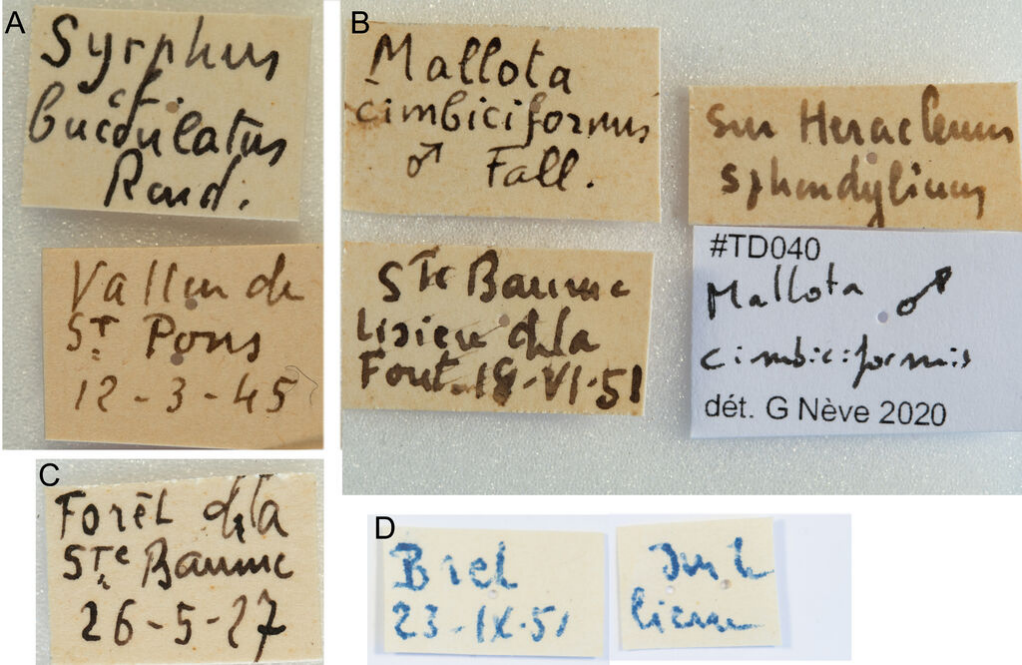
Examples of labels from the Timon-David Collection. A: specimen MHNM.15441.47.135, collected at Vallon de St-Pons, Gemenos, Bouches-du-Rhône, now identified as *Eupeodes luniger*; B: specimen MHNM.15441.48.111, collected on the edge of the Ste Baume forest, Plan-d'Aups, Var, on *Heracleum sphondylium*, identification as *Mallota cimbiciformis* confirmed in 2020; C: specimen MHNM.15441.40.1, collected in Ste Baume forest, Plan-d'Aups, Var, now identified as* Myolepta dubia*; D: specimen MHNM.15441.43.117, collected at Bret, Le Tholonet, Bouches-du-Rhône, on *Hedera helix* ("sur le lierre"), now identified as *Merodon legionensis*.

All available data on the 1071 hoverfly specimens present in the Timon-David collection in the Natural History Museum in Marseille were input in table format and made available on Zenodo (https://zenodo.org/doi/10.5281/zenodo.10362019). Labels of specimens collected by Jean Timon-David were always hand-written and usually mention the locality and a date, sometimes with the plant name on which the specimen was collected (Fig. 3). Jean Timon-David never put his own name on the specimen labels.

For most of the specimens, their identification was checked by Gabriel Nève (GN), Xavier Lair (XL) and Thomas Lebard (TL) and this is mentioned with the value 1 in the identificationVerificationStatus column, which is otherwise coded 0 for unchecked material. All label data from the Syrphidae specimens were input and most of the French specimens were re-identified according to recent revisions (e.g. Speight and Sarthou 2017, Speight and Langlois 2020, Speight et al. 2021). Latitudes and longitudes of all data from France were input using the topographical maps on the www.geoportail.gouv.fr website or printed 1/25000 maps of the studied areas. Data with label localities which corresponded to municipalities were input on the locality’s centroid in latitude and longitude columns and their estimated uncertainty value (coordinateUncertaintyValue column) was set at 5000 metres. Data with more precise names were identified and input with an estimated uncertainty of 1000 metres. Data from countries other than France were usually far less precise; these were located using googleEarthPro and input with either a 5 km or 50 km uncertainty value. If a large administrative area only was mentioned on the label, such as Lafayette County, Miss., then a larger coordinate uncertainty was mentioned. Two locations could not be located and their coordinates are missing. If no location label was present under the specimen, verbatimLocality was given as missing data (“[non renseigné]”).

The whole dataset was then formatted into a GBIF compatible file, which retains the original identifications, as well as the revised identification (if any).

In a few cases, the specimen could be identified only to a species group. This is indicated with “cf.” in identificationQualifier column, with one species of the group mentioned in scientificName. In three cases, the identification was only possible within a pair of species and the two species were mentioned in identificationRemarks: *Microdon mutabilis* or *Microdon myrmicae* (Schönrogge et al. 2002), *Merodon moenium* or *Merodon avidus* (Speight and Langlois 2020) and *Cheilosia albitarsis* or *Cheilosia ranunculi* (Doczkal 2000).

### Step description

All data on specimen labels of the Timon-David Syrphidae collection were encoded in a table format. Every specimen received an individual label with its inventory Museum number. A total of 756 French hoverfly specimens were re-identified by GN, XL or TL. If the original identification had to be changed, the former identification was mentioned in the PreviousIdentification column and the updated one in the ScientificName column and the value in the identificationVerificationStatus column set as 1. The taxonomy of European species follows the list used by Vujić et al. (2022) for the Red List of European hoverflies. The taxonomy of specimens from other continents was checked with the Systema Dipterorum website (Evenhuis and Pape 2023). If the name had to be changed, but the identification of the specimen could not be checked, the updated name is mentioned in the scientificName column and a 0 value was put in the identificationVerificationStatus column.

There is only one paratype in the whole collection: an Australian specimen of *Sphiximorpha alaplicata* (Hardy 1945); in this case, typeStatus was set as “paratype” and its identificationVerificationStatus status was set as 1.

The type of *Cheilosia vangaveri *Timon-David, 1937 could not be located, despite Timon-David (1937a) mentioning specifically that the type remained in his collection. It was last seen in 1974 by E. Thorpe, but its subsequent whereabouts could not be traced (Barkalov and Ståhls 1997). Unfortunately, there is no surviving correspondence regarding Timon-David’s collection which could help (P. Timon-David, pers. comm. 2023).

Occurrence remarks: Here, ecological data are indicated, if any (host plant, altitude or other information, such as a record number given by Timon-David, regarding the specimen). A total of 72 French specimen labels only include numbers and these were probably taken during Timon-David’s studies on the islands of Marseille (Van Gaver and Timon-David 1929a, Timon-David 1940, Timon-David 1961) or the Alps (Timon-David 1937a); as their capture data are lacking, they could not be assigned to a specific location, only to France.

## Geographic coverage

### Description

**Figure 4.**
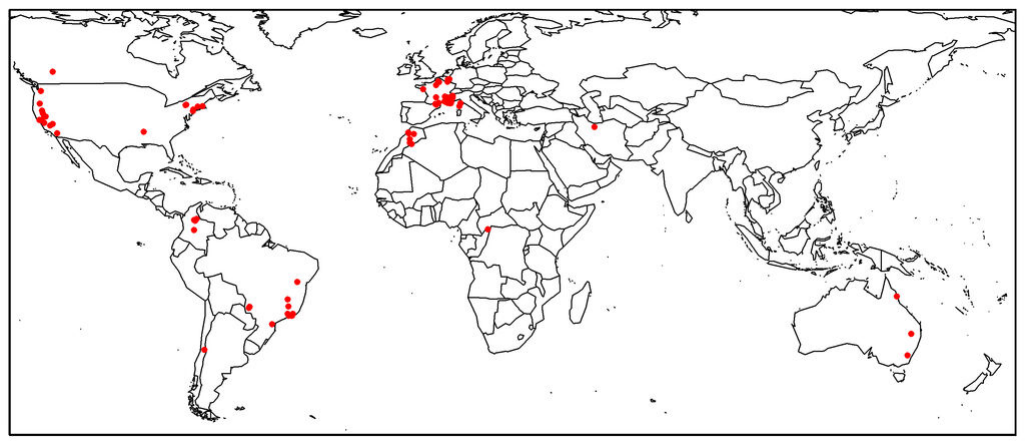
World distribution of hoverfly specimens in the collection of Jean Timon-David.

**Figure 5.**
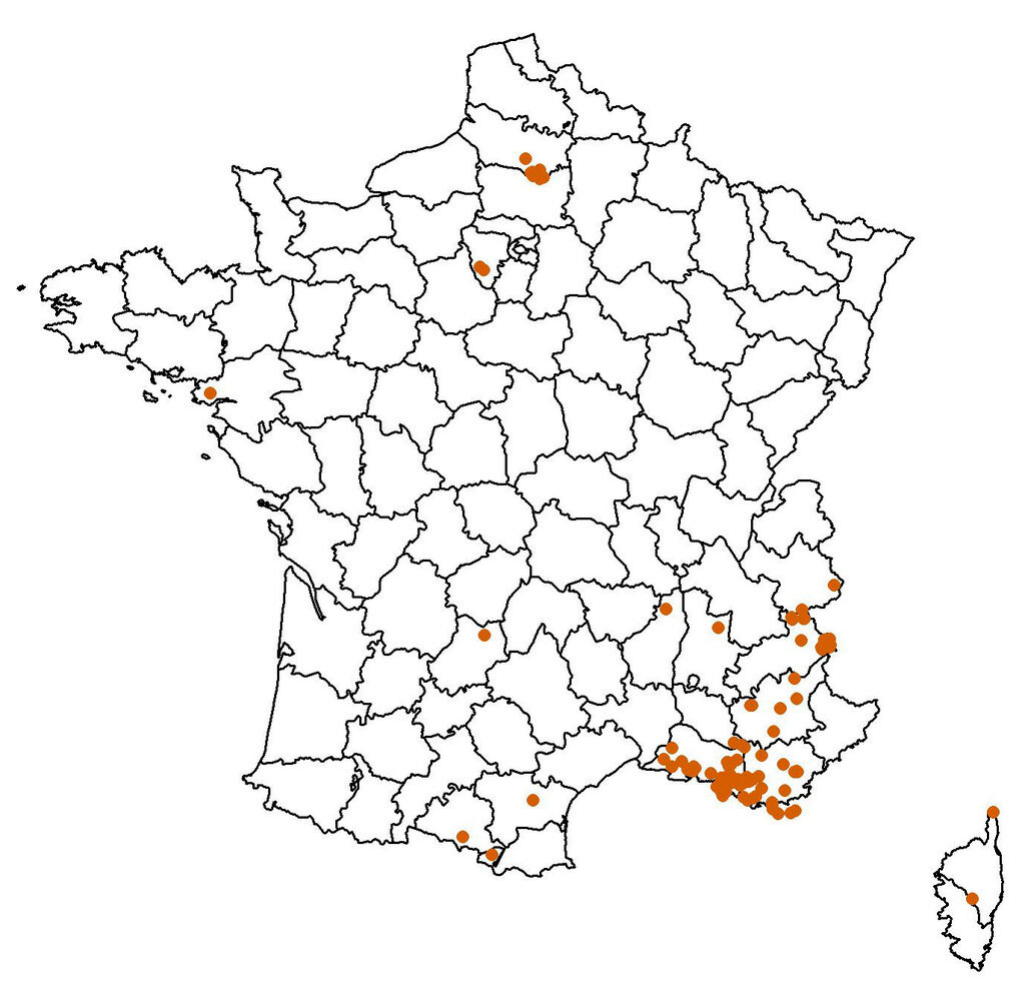
Distribution of French specimens of hoverflies in the collection of Jean Timon-David.

Worldwide (Fig. 4; Table 1); 89% of the data from France (Table 2, Fig. 5).

### Coordinates

-36 and 53 Latitude; -123 and 151 Longitude.

## Taxonomic coverage

### Description

Specimens of 239 named species and of one named subspecies belonging to the family Syrphidae (Table 3).

## Temporal coverage

**Data range:** 1896-5-10 – 1961-7-04.

## Collection data

### Collection name

Timon-David collection

### Collection identifier

MNHN.15441

### Parent collection identifier

Insects

### Specimen preservation method

Dried and pinned specimens

### Curatorial unit

Muséum d’Histoire Naturelle de Marseille (MHNM), contact: Christophe Borrely (email: cborrely@marseille.fr)

## Usage licence

### Usage licence

Creative Commons Public Domain Waiver (CC-Zero)

### IP rights notes

IP rights notes: This work is licensed under a Creative Commons Attribution (CC-BY) 4.0 Licence. All work derived from the present study should cite it appropriately, including the Museum where the material is held.

## Data resources

### Data package title

Syrphidae in the Jean Timon-David collection, Marseille

### Resource link

https://zenodo.org/records/10362020

### Alternative identifiers

https://zenodo.org/doi/10.5281/zenodo.10362019

### Number of data sets

1

### Data set 1.

#### Data set name

Timon-David Syrphidae collection

#### Data format

CSV (tab delimited values)

#### Character set

TimonDavidSyrphidaeColl_v01.csv

#### Data format version

Darwin core, so that it could be transferred later into GBIF as more identifications are checked.

#### Description

The dataset includes data on 1071 specimens of Syrphidae collected or received by Jean Timon-David, in GBIF compatible format.

**Data set 1.** 

Column label	Column description
occurrenceID	Individual identification code: same as CatalogNumber
catalogNumber	MHNM individual identification: combination of Museum name, collection identification, box number and specimen number within each box
basisOfRecord	The specific nature of the data record (i.e. PreservedSpecimen)
eventDate	Event date in the format YYYY-MM-DD if the capture date is known to the date, or YYYY-MM if only the month and year are known, or YYYY if only the year is known
year	Year of capture if known
month	Month of capture if known
day	Day of capture if known
verbatimEventDate	Date of capture, as mentioned on the label
scientificName	Lowest taxonomic rank possible, usually the species name. If unknown, the genus or family names are given
identificationQualifier	In case the identification could be given only to a species group 'cf.' is input
identificationRemarks	Any comment on the identification of the specimen
kingdom	Kingdom (i.e. Animalia)
phylum	Phylum (i.e. Arthropoda)
class	Class (i.e. Insecta)
order	Order (i.e. Diptera)
family	Family name (i.e. Syrphidae)
genus	Genus name
specificEpithet	Species epithet of the scientificName
sex	Male (M) or female (F)
taxonRank	Taxonomic rank of the most specific name in the scientificName
identifiedBy	Name of the entomologist who identified the specimen. The name is written within square brackets if it does not appear on the label, but can be inferred from other specimens with similar handwriting, locality and date
dateIdentified	Year of identification
identificationVerificationStatus	Whether (coded 1) or not (coded 0) the identification was recently checked
previousIdentifications	Species name originally given on the specimen labels
decimalLatitude	Geographic latitude (in decimal degrees) of the capture location
decimalLongitude	Geographic longitude (in decimal degrees) of the capture location
geodeticDatum	Coordinate system and set of reference points upon which the geographic coordinates are based (i.e. WGS 84)
coordinateUncertaintyInMetres	Uncertainty in coordinates, in metres
continent	Continent of capture
country	Country of capture
countryCode	Two letter country code of the specimen origin
stateProvince	French Departmental administrative division. In the case of non-French data, any relevant country administrative subdivision
locality	Location of capture, usually the locality
verbatimLocality	Any geographical indication on the label
InstitutionCode	Museum where the specimen is held (i.e. MHNM)
occurrenceRemarks	Any ecological data or comment on the label
LocationRemarks	Any comment regarding the location
recordedBy	Name of collector (i.e. *legit* information)
associatedRereferences	Any reference citing the specimen
organismQuantity	Number of individuals bearing the same label (usually 1)
organismQuantityType	Individuals
georeferencedBy	Identity of the person who added the Latitude and longitude data, i.e. Nève, Gabriel
georeferenceProtocol	How the georeference was computed, i.e. from label data (verbatimLocality)
georeferenceSources	Georeference code was inferred from geoportail.fr, French ING maps or googleEarthPro
georeferencedDate	Georeference work was performed in 2023
language	The dataset is mainly written in French, apart from column headings, which are in English
collectionCode	Identifier of collection (i.e. MNHN.15441)
otherCatalogueNumbers	Any other catalogue number the specimen may have
typeStatus	One specimen is a paratype, which is indicated here as such.
minimumElevationInMetres	Lower limit of the range of altitudes indicated on the label or in the associated reference.
maximumElevationInMetres	Higher limit of the range of altitude indicated on the label or in the associated reference

## Additional information

### Abbreviation used throughout

MHNM: Muséum d’Histoire Naturelle de Marseille (Bouches-du-Rhône, France)

### Publishing organisation

Muséum d’Histoire Naturelle de Marseille (MHNM)

### Museum identifier

MHNM (Muséum d'Histoire Naturelle de Marseille)

### Contact

MHNM: Christophe Borrely, cborrely@marseille.fr

### Dataset management

Gabriel Nève: gabriel.neve@imbe.fr

### General discussion

The Syrphidae collected by Timon-David or received by him total 1071 specimens, mainly from France, but also from the Americas, Africa and Australia (Fig. 4). Unfortunately, the type specimen of *Cheilosia vangaveri* Timon-David 1937 could not be found in his collection. He probably lent it to a colleague whom we have been unable to identify.

Timon-David always retained a particular interest in the Sainte-Baume massif (Var), from which 155 hoverflies in his collection were collected between 1927 and 1959 (Timon-David 1936, Timon-David 1958). Another locality with a large number of collected specimens is his property at La Viste, a suburb of Marseille, with 118 records between 1926 and 1945. Timon-David left this property at the end of the 1940s when the northern motorway of Marseille was built. He then regularly visited the family property at Le Tholonet, near Aix-en-Provence (32 records from 1941 to 1952) and, in particular, the hamlet of Bret, where he collected 25 specimens from 1950 to 1954. He was interested in the entomological fauna of the Marseille islands (Timon-David 1940, Timon-David 1961), where his data remain the only ones on Diptera. He always hunted by sight, using an entomological net, which explains the rarity of small species (genera *Paragus, Orthonevra* etc.) in his collection.

Some of the data from his collection have never been published and some of the specimens remained unidentified. We have now addressed this issue for all specimens originating from the Bouches-du-Rhône and Var Departments. His collection now contains 756 French specimens with reliable species identification labels.

The examination of this collection improves our knowledge of the distribution of French Syrphidae species. Compared with the most recent national database (Speight et al. 2018, Speight et al. 2020) and recent additions to departmental data (Nève 2018, Ropars et al. 2020, Lebard 2022, Solère et al. 2022), Timon-David’s collection adds 34 species to the known hoverfly faunas of the Bouches-du-Rhône and 12 species to the Var Department, among others, leading to a total of 85 new Departmental hoverfly records (Table 4).

**Figure 6.**
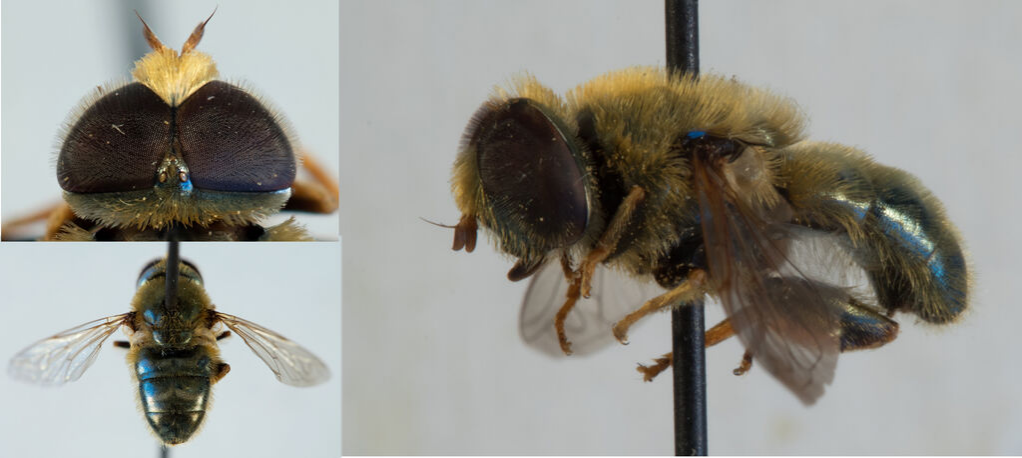
Habitus and details of head and abdomen of a specimen of *Merodon legionensis* collected at Bret, Le Tholonet, Bouches-du-Rhône, France, on 23 September 1951, the oldest French record of this species.

Among the added species noteworthy is the oldest record of *Merodon legionensis *(Fig. 6) for France (Louboutin and Speight 2021).

Four species in the collection are classified as endangered at the European level (Vujić et al. 2022): *Cheilosia venosa*, *Chrysotoxum gracile*, *Epistrophe leiophthalma* and *Eumerus hungaricus*, five as vulnerable (*Callicera rufa*, *Callicera spinolae*, *Microdon mutabilis*, *Pipizella brevis* and *Spilomyia digitata*) and eight as near threatened (*Cheilosia marginata*, *Chrysotoxum elegans*, *Chrysotoxum octomaculatum*, *Merodon flavus*, *Merodon legionensis*, *Merodon unicolor*, *Microdon analis* and *Spazigaster ambulans*).

Timon-David's collection dataset will be used as one of the key sources for documenting the status of the south-eastern French hoverfly fauna present during the twentieth century, particularly in the Mediterranean area, with the hope that the recorded species will continue to thrive in the studied area.

## Figures and Tables

**Table 1.** Number of Syrphidae in the Timon-David collection, sorted by country of origin.

**Country**	**Number of specimens**
Australia	4
Belgium	3
Brazil	42
Canada	1
Central African Republic	7
Chile	1
Colombia	6
France	951
Iran	1
Morocco	9
United States of America	45
[Unknown]	1
Total	1071

**Table 2.** Number of Syrphidae from France in the Timon-David collection, by French Department.

**French Department**	**Number of specimens**
Alpes-de-Haute-Provence	32
Ardèche	19
Ariège	68
Bouches-du-Rhône	362
Drôme	18
Haute-Corse	3
Haute-Savoie	51
Hautes-Alpes	62
Loire-Atlantique	8
Lot	2
Oise	1
Pyrénées-Orientales	2
Savoie	4
Somme	23
Var	220
Vaucluse	1
Yvelines	3
[Unknown]	72
Total	951

**Table 3.** Number of specimens by species in the Timon-David Syrphidae collection.

**Species**	**Unverified**	**Verified**
*Allograpta obliqua* (Say, 1823)	1	0
*Arctophila flagrans* Osten Sacken, 1875	1	0
*Asemosyrphus polygrammus* (Loew, 1872)	1	0
*Baccha clavata* (Fabricius, 1794)	1	0
*Baccha crocata* Austen, 1893	1	0
*Baccha dimidiata* (Fabricius, 1781)	1	0
*Baccha elongata* (Fabricius, 1775)	5	2
*Baccha lepida* Macquart, 1842	1	0
*Baccha livida* Schiner, 1868	1	0
*Blera fallax* Linnaeus, 1758	0	1
*Brachyopa scutellaris* (Robineau-Devoisy in Desmarest, 1843)	0	2
*Brachypalpoides lentus* (Meigen, 1822)	0	10
*Brachypalpus laphriformis* (Fallén, 1816)	0	4
*Brachypalpus margaritus* Hull, 1946	1	0
*Caliprobola speciosa* (Rossi, 1790)	0	6
*Callicera marcquarti* (Rondani, 1844)	0	2
*Callicera rufa* (Schummel, 1842)	0	1
*Callicera spinolae* Rondani, 1844	0	1
*Ceriana conopsoides* Linnaeus, 1758	0	1
*Ceriana opuntiae* (Ferguson, 1926)	1	0
*Ceriana ornata* (Saunders, 1845)	1	0
*Ceriana vespiformis* (Latreille, 1804)	0	1
*Cheilosia albipila* (Meigen, 1838)	0	1
*Cheilosia albitarsis* (Meigen, 1822)	0	16
*Cheilosia barbata* Loew, 1857	2	0
*Cheilosia brunnipennis* (Becker, 1894)	0	1
*Cheilosia caerulescens* (Meigen, 1822)	2	0
*Cheilosia canicularis* (Panzer, 1801)	3	0
*Cheilosia chloris* (Meigen, 1822)	1	0
*Cheilosia chrysocoma* (Meigen, 1822)	1	0
*Cheilosia derasa* Loew, 1857	1	0
*Cheilosia gigantea* (Zetterstedt, 1838)	1	0
*Cheilosia grossa* (Fallén, 1817)	2	3
*Cheilosia illustrata* (Harris, 1776)	4	0
*Cheilosia impressa* Loew, 1840	1	1
*Cheilosia marginata* Becker, 1894	1	0
*Cheilosia* sp.	3	0
*Cheilosia melanura* Becker, 1894	1	0
*Cheilosia mutabilis* (Fallén, 1817)	1	4
*Cheilosia pagana* (Meigen, 1822)	1	2
*Cheilosia proxima* (Zetterstedt, 1843)	1	0
*Cheilosia ranunculi* Dockzal, 2000	0	9
*Cheilosia scutellata* (Fallén, 1817)	2	2
*Cheilosia soror* (Zetterstedt, 1843)	0	14
*Cheilosia urbana* (Meigen, 1822)	0	11
*Cheilosia variabilis* (Panzer, 1798)	4	0
*Cheilosia venosa* Loew, 1857	1	0
*Cheilosia vernalis* (Fallén, 1817)	2	0
*Cheilosia vulpina* (Meigen, 1822)	0	1
*Chrysogaster solstitialis* (Fallén, 1817)	0	1
*Chrysotoxum bicinctum* (Linnaeus, 1758)	0	9
*Chrysotoxum cautum* (Harris, 1776)	0	14
*Chrysotoxum elegans* Loew, 1841	0	2
*Chrysotoxum fasciatum* (Müller, 1764)	0	5
*Chrysotoxum fasciolatum* (De Geer, 1776)	0	5
*Chrysotoxum festivum *(Linnaeus, 1758)	0	6
*Chrysotoxum gracile* (Becker, 1921)	0	1
*Chrysotoxum intermedium* Meigen, 1822	0	26
*Chrysotoxum octomaculatum* Curtis, 1837	0	1
*Chrysotoxum vernale* Loew, 1841	0	5
*Copestylum azureum* (Philippi, 1865)	1	0
*Copestylum trituberculatum* (Thompson, 1976)	2	0
*Criorhina asilica* (Fallén, 1816)	0	1
*Criorhina berberina* (Fabricius, 1805)	0	4
*Criorhina floccosa* (Meigen, 1822)	0	2
*Criorhina ranunculi* (Panzer, 1804)	0	1
*Cynorhina analis* (Macquart, 1842)	1	0
*Cynorhina scitula* Williston, 1882	1	0
*Dasysyrphus albostriatus* (Fallén, 1817)	1	7
*Dasysyrphus tricinctus* (Fallén, 1817)	0	1
*Didea fasciata* Macquart, 1834	0	3
*Doros profuges* (Harris, 1780)	0	1
*Epistrophe caldus* Walker, 1852	1	0
*Epistrophe eligans* (Harris, 1780)	0	4
*Epistrophe eligans trifasciata* Gomes, 1978	0	9
*Epistrophe leiophthalma* (Schiner & Egger, 1853)	4	1
*Epistrophe nitidicollis* (Meigen, 1822)	2	3
*Epistrophella euchroma* Kowarz, 1885	0	2
*Episyrphus balteatus* (de Geer, 1776)	2	10
Eristalini	6	0
*Eristalinus aeneus* (Scopoli, 1763)	2	10
*Eristalinus *sp.	0	5
*Eristalinus sepulchralis* (Linnaeus, 1758)	0	6
*Eristalinus taeniops* (Wiedemann, 1818)	1	7
*Eristalis arbustorum* (Linnaeus, 1758)	1	3
*Eristalis fasciatus* Wiedemann, 1819	1	0
*Eristalis jugorum* Egger, 1858	2	0
*Eristalis *sp.	4	4
*Eristalis melanaspis* Wiedemann, 1830	1	0
*Eristalis nemorum* (Poda, 1761)	1	0
*Eristalis obsoletus* Wiedemann, 1830	1	0
*Eristalis pertinax* (Scopoli, 1763)	0	1
*Eristalis pygolampus* Wiedemann, 1830	1	0
*Eristalis rupium* Fabricius, 1805	3	0
*Eristalis tenax* (Linnaeus, 1758)	5	11
*Eumerus alpinus* (Rondani, 1857)	0	1
*Eumerus amoenus* Loew, 1848	0	1
*Eumerus barbarus* (Coquebert, 1804)	0	1
*Eumerus hungaricus* (Szilady, 1940)	0	1
*Eumerus *sp.	1	10
*Eumerus nudus* (Loew, 1848)	0	2
*Eumerus ornatus* Meigen, 1822	0	2
*Eumerus strigatus* (Fallén, 1817)	1	0
*Eumerus tricolor* (Fabricius, 1898)	0	1
*Eupeodes bucculatus* (Rondani, 1857)	0	1
*Eupeodes corollae* (Fabricius, 1794)	1	8
*Eupeodes flaviceps* (Rondani, 1857)	3	0
*Eupeodes latifasciatus* Macquart, 1829	0	1
*Eupeodes luniger* (Meigen, 1822)	1	2
*Eupeodes volucris* Osten Sacken, 1877	1	0
*Ferdinandea aurea* (Rondani, 1844)	0	1
*Ferdinandea cuprea* (Scopoli, 1763)	0	7
*Ferdinandea dives* (Osten Sacken, 1877)	1	0
*Helophilus *sp.	0	1
*Helophilus pendulus* (Linnaeus, 1758)	0	2
*Helophilus trivittatus* (Fabricius, 1805)	2	7
*Heringia heringi* (Zetterstedt, 1843)	0	7
*Hybobathus flavipennis* (Wiedemann, 1830)	1	0
*Ischiodon aegyptius* (Wiedemann, 1830)	1	0
*Lapposyrphus lapponicus* Zetterstedt, 1838	0	1
*Lejogaster metallina* (Fabricius, 1777)	0	1
*Mallota cimbiciformis* (Fallén, 1817)	1	1
*Mallota posticata* (Fabricius, 1805)	1	0
*Melangyna barbifrons* (Fallén, 1817)	1	0
*Melangyna compositarum* Verrall, 1873	0	5
*Melangyna umbellatarum* (Fabricius, 1794)	0	3
*Melanostoma dubium* (Zetterstedt, 1838)	0	1
*Melanostoma mellinum* (Linnaeus, 1758)	1	7
*Melanostoma scalare* (Fabricius, 1794)	0	6
*Melanostoma *sp.	1	0
*Meligramma triangulifera* (Zetterstedt, 1843)	0	1
*Meliscaeva *sp.	0	1
*Meliscaeva auricollis* (Meigen, 1822)	2	15
*Meliscaeva cinctella* Zetterstedt, 1843	1	0
*Merodon albifrons* (Meigen, 1822)	0	14
*Merodon aureus* Fabricius, 1805	1	4
*Merodon avidus* (Rossi, 1790)	0	12
*Merodon cinereus* (Fabricius, 1794)	0	3
*Merodon clavipes* (Fabricius, 1781)	0	38
*Merodon elegans* (Hurkmans, 1993)	0	3
*Merodon equestris* Fabricius, 1794	0	19
*Merodon flavus* (Sack, 1913)	0	4
*Merodon funestus* Fabricius, 1794	0	1
*Merodon legionensis* Marcos-Garcia, Vujic & Mengual, 2007	0	1
*Merodon *sp.	0	1
*Merodon moenium* Wiedemann, 1822	0	7
*Merodon nigritarsis* (Rondani, 1845)	0	3
*Merodon obscuritarsis* (Strobl, 1809)	0	2
*Merodon serrulatus* (Wiedemann in Meigen, 1822)	0	7
*Merodon spicatus* Becker, 1907	1	0
*Merodon unicolor* (Strobl, 1909)	0	2
*Meromacrus acutus* (Fabricius, 1805)	1	0
*Mesograpta marginatum* (Say, 1823)	1	0
*Microdon *sp.	6	0
*Microdon analis* (Macquart, 1842)	0	2
*Microdon luteiventris* Bezzi, 1915	1	0
*Microdon mutabilis* (Linnaeus, 1758)	0	1
*Milesia crabroniformis* (Fabricius, 1775)	0	9
*Milesia semiluctifera* (Villers, 1789)	0	20
*Milesia virginiensis* (Drury, 1773)	1	0
*Myathropa florea* (Linnaeus, 1758)	0	12
*Myolepta dubia* Fabricius, 1802	0	16
*Myolepta potens* (Harris, 1780)	0	3
*Myolepta vara* (Panzer, 1798)	0	2
*Neoascia annexa* (Müller, 1776)	0	1
*Neoascia podagrica* (Fabricius, 1775)	0	5
*Ocyptamus funebris* Macquart, 1834	1	0
*Ornidia obesa* (Fabricius, 1775)	2	0
*Palpada agrorum* (Fabricius, 1787)	1	0
*Palpada furcata* (Chr. Wiedemann, 1819)	1	0
*Palpada triangularis* (Gilgio-Tos, 1882)	1	0
*Palpada vinetorum* (Fabricius, 1798)	1	0
*Paragus bicolor* (Fabricius, 1794)	1	5
*Paragus haemorrhous* Meigen, 1822	0	1
*Paragus *sp.	0	7
*Paragus quadrifasciatus* (Meigen, 1822)	0	2
*Paragus tibialis* (Fallén, 1817)	0	1
*Parasyrphus annulatus* (Zetterstedt, 1838)	0	1
*Parhelophilus frutetorum* (Fabricius, 1775)	0	1
*Pipiza austriaca* Meigen, 1822	1	0
*Pipiza *sp.	0	1
*Pipiza festiva* Meigen, 1822	0	12
*Pipiza lugubris* (Fabricius, 1775)	1	0
*Pipiza quadrimaculata* (Panzer, 1804)	2	0
*Pipizella brevis* (Lucas, 1977)	0	1
*Pipizella *sp.	0	6
*Pipizella viduata* (Linnaeus, 1758)	0	6
*Pipizella virens* (Fabricius, 1805)	4	0
*Platycheirus albimanus* (Fabricius, 1781)	2	1
*Platycheirus ambiguus* (Fallén, 1817)	2	1
*Platycheirus peltatus* (Meigen, 1822)	1	0
*Platycheirus scutatus* (Meigen, 1822)	0	1
*Pterallastes thoracicus* (Loew, 1863)	1	0
*Rhingia campestris* (Meigen, 1822)	1	0
*Rhingia rostrata* (Linnaeus, 1758)	4	0
*Salpingogaster niger* Schiner, 1868	1	0
*Scaeva dignota* (Rondani, 1857)	0	5
*Scaeva *sp.	0	1
*Scaeva pyrastri* (Linnaeus, 1758)	0	12
*Scaeva selenitica* (Meigen, 1822)	4	2
*Serichlamys mitis* (Curran, 1940)	2	0
*Sericomyia bombiformis* (Fallén, 1810)	0	4
*Sericomyia chalcopyga* Loew, 1863	1	0
*Sericomyia chrysotoxoides* Macquart, 1842	1	0
*Sericomyia lappona* (Linnaeus, 1758)	0	1
*Sericomyia militaris* Walker, 1849	1	0
*Sericomyia silentis* (Harris, 1776)	0	4
*Sericomyia superbiens* Müller, 1776	0	4
*Somula decora* Macquart, 1847	1	0
*Spazigaster ambulans* (Fabricius, 1798)	1	0
*Sphaerophoria interrupta* (Fabricius, 1805)	1	0
*Sphaerophoria* Le Peletier & Audinet-Serville, 1828	2	1
*Sphaerophoria menthastri* (Linnaeus, 1758)	1	0
*Sphaerophoria rueppelli* (Wiedemann, 1830)	0	3
*Sphaerophoria scripta* (Linnaeus, 1758)	6	9
*Sphaerophoria taeniata* (Meigen, 1822)	3	0
*Sphegina clunipes* (Fallén, 1816)	2	0
*Sphegina elegans* Schummel, 1843	0	1
*Sphiximorpha alaplicata* (Hardy, 1945)	0	1
*Sphiximorpha breviscapa* (Saunders, 1845)	1	0
*Spilomyia digitata* Rondani, 1865	0	8
*Spilomyia longicornis* (Loew, 1872)	1	0
*Spilomyia saltuum* (Fabricius, 1794)	0	1
*Syritta pipiens* (Linnaeus, 1758)	6	4
Syrphidae	93	0
*Syrphus* sp.	2	0
*Syrphus ribesii* (Linnaeus, 1758)	0	10
*Syrphus torvus* Osten-Sacken, 1875	3	2
*Syrphus vitripennis* (Meigen, 1822)	1	7
*Temnostoma balyras* (Walker, 1849)	1	0
*Temnostoma pictulum* Williston, 1887	1	0
*Temnostoma vespiforme* (Linnaeus, 1758)	0	1
*Toxomerus boscii* (Macquart, 1842)	1	0
*Toxomerus geminatus* (Say, 1823)	1	0
*Toxomerus jussiaeae* Vigé, 1939	1	0
*Toxomerus politus* (Say, 1823)	2	0
*Toxomerus tibicen* (Chr. Wiedemann, 1830)	2	0
*Tropidia albistylum* Macquart, 1847	1	0
*Tropidia quadrata* (Say, 1824)	1	0
*Tropidia scita* (Harris, 1780)	1	0
*Volucella bombylans* (Linnaeus, 1758)	0	8
*Volucella inanis* (Linnaeus, 1758)	0	5
*Volucella inflata* (Fabricius, 1794)	0	8
*Volucella liquida* Erichson, 1841	2	0
*Volucella pellucens* (Linnaeus, 1758)	0	10
*Volucella picta* Wiedemann, 1830	1	0
*Volucella scutellata* Macquart, 1842	2	0
*Volucella zonaria* (Poda, 1761)	0	14
*Xanthandrus bucephalus* (Chr. Wiedemann, 1830)	1	0
*Xanthandrus comtus* (Harris, 1776)	0	2
*Xanthogramma citrofasciatum* (De Geer, 1776)	1	2
*Xanthogramma dives* (Rondani, 1857)	0	11
*Xanthogramma maculipenne* Mik, 1887	1	0
*Xanthogramma pedissequum* (Harris, 1776)	0	4
*Xanthogramma stackelbergi* Violovitsh, 1975	0	1
*Xylota segnis* (Linnaeus, 1758)	0	9
*Xylota sylvarum* (Linnaeus, 1758)	0	3

**Table 4.** Additions to known French Departmental fauna.

French Departments	Species added to each Departmental known fauna
Alpes-de-Haute-Provence	*Merodon aureus*
	*Microdon mutabilis*
Ardèche	*Callicera rufa*
	*Chrysotoxum fasciolatum*
	*Neoascia annexa*
	*Sphegina elegans*
Ariège	*Chrysogaster solstitialis*
	*Chrysotoxum bicinctum*
	*Doros profuges*
	*Merodon equestris*
	*Sericomyia superbiens*
	*Volucella inanis*
	*Volucella pellucens*
	*Xanthogramma dives*
	*Xanthogramma stackelbergi*
	*Xylota segnis*
Bouches-du-Rhône	*Baccha elongata*
	*Caliprobola speciosa*
	*Ceriana conopsoides*
	*Cheilosia albipila*
	*Cheilosia albitarsis*
	*Cheilosia impressa*
	*Cheilosia pagana*
	*Cheilosia urbana*
	*Chrysotoxum bicinctum*
	*Criorhina floccosa*
	*Eristalinus taeniops*
	*Eumerus amoenus*
	*Eumerus barbarus*
	*Eumerus hungaricus*
	*Eumerus nudus*
	*Eumerus ornatus*
	*Eumerus tricolor*
	*Eupeodes bucculatus*
	*Ferdinandea aurea*
	*Ferdinandea cuprea*
	*Merodon avidus*
	*Merodon elegans*
	*Merodon equestris*
	*Merodon legionensis*
	*Milesia crabroniformis*
	*Milesia semiluctifera*
	*Pipizella viduata*
	*Platycheirus ambiguus*
	*Platycheirus scutatus*
	*Scaeva dignota*
	*Spilomyia digitata*
	*Spilomyia saltuum*
	*Volucella inanis*
	*Xylota segnis*
Drôme	*Chrysotoxum elegans*
	*Chrysotoxum gracile*
	*Epistrophe leiophthalma*
	*Merodon cinereus*
	*Scaeva dignota*
	*Syrphus torvus*
	*Xylota segnis*
Haute-Corse	*Merodon equestris*
Hautes-Alpes	*Chrysotoxum fasciolatum*
	*Chrysotoxum festivum*
	*Merodon aureus*
	*Sericomyia bombiformis*
	*Syrphus torvus*
Loire-Atlantique	*Lejogaster metallina*
Lot	*Caliprobola speciosa*
	*Criorhina asilica*
Savoie	*Platycheirus ambiguus*
Somme	*Dasysyrphus tricinctus*
	*Melangyna compositarum*
	*Melangyna umbellatarum*
	*Xylota sylvarum*
Var	*Brachyopa scutellaris*
	*Cheilosia grossa*
	*Cheilosia pagana*
	*Cheilosia vulpina*
	*Criorhina ranunculi*
	*Epistrophella euchroma*
	*Melangyna umbellatarum*
	*Merodon elegans*
	*Pipiza festiva*
	*Platycheirus albimanus*
	*Xanthogramma pedissequum*
	*Xylota segnis*
Vaucluse	*Chrysotoxum intermedium*
